# Nutritional quality, nutrient uptake and biomass production of *Pennisetum purpureum* cv. King grass

**DOI:** 10.1038/s41598-021-93301-w

**Published:** 2021-07-05

**Authors:** Julián M. Botero-Londoño, Erika M. Celis-Celis, Mónica A. Botero-Londoño

**Affiliations:** 1 Universidad Industrial de Santander, Málaga, Colombia; 2 Universidad Industrial de Santander, Bucaramanga, Colombia

**Keywords:** Plant sciences, Plant development, Plant physiology

## Abstract

The research was conducted to determine the effects of cutting interval and fertilization on the nutritional quality, nutrient uptake, and biomass production of King grass. The experimental design was a randomized complete block, using 4 blocks and 8 treatments per block; treatments consisted of 4 ages of cutting (30, 45, 60, and 90 days), with fertilization and without fertilization. The results showed increases of up to 72,000 kg ha^−1^ year^−1^ of dry matter (DM) when fertilization was implemented. There was a significant reduction in with an increase in the cutting days (12.70–6.53% protein). Fiber increased (48.79–72.99% NDF) when fertilization treatments were included and cutting days increased. The elements that were included in fertilization (N, P, K) showed a higher foliar content and also presented a reduction in foliar content with growth of the plant. Treatments with fertilization showed a nutrient uptake increase for all the elements up to 60 days, where a reduction in uptake capacity was observed. King grass is a plant with a high nutrient uptake capacity and, therefore, with high biomass and nutrient production. This is an advantage since it can be used in multiple applications, such as animal feed, biofuel production, and as a substrate for biodigestion, among others.

## Introduction

In tropical regions, cattle farming bases its feed on the production of pastures and forage because of its low cost of production and availability^1,2^. In this context, varieties of *Pennisetum purpureum* are highly used as an animal feed alternative mainly in the dry seasons, due to high biomass production and as a strategy to complement grazing so as to increase the load capacity and fill the shortage of quality pastures^3^.

King grass’s (*Pennisetum purpureum)* high biomass production has positioned it as an important forage material for renewable energy production^4^. Gallego determined that King grass is one of the plants with the greatest potential for butanol production thanks to its productive characteristics, biomass production, and solvent yield^5^. Cardona classified it as a high-performance perennial grass with the capacity to adapt to various soil conditions and a high potential for ethanol production, even showing higher yields than sugarcane bagasse^6^.

Ordaz evaluated the nutritional composition of King grass at different cutting intervals (90, 120, 150, and 180 days), finding that as the cutting days increased, fiber levels also increased and crude protein levels and digestibility of DM significantly decreased. Additionally, they found that the maximum values of crude protein in leaf were 10.1% at 90 cutting days and decreased to 5.6% at 180 days^7^.

Biomass production and nutrient concentration in forage is determined by its availability in soils, therefore, the interpretation of soil analyses and the application of fertilizers based on this interpretation is a determining factor in crop development^8–11^. Consequently, studies should be carried out in each production area related to the use of fertilizers for the purpose of maximizing plant productivity based on agroclimatic and soil characteristics to obtain models that maximize yield^12^.

This research determined biomass production, nutrient concentration, and nutrient uptake from King grass based on cutting days and fertilization.

## Methods

The research was developed at La Esmeralda farm located in the municipality of Circasia Quindío, in the Colombian coffee region, located at 4° and 38′ 24″ north latitude and 75° and 38′ 26″ west longitude at an elevation of 1660 m, with an annual rainfall of 2400 mm and an average temperature of 19 °C. The study was carried out in an Andisol soil with an acidic pH (5.4–5.6), high organic matter content (8.6–8.9%), high cation exchange capacity (21.3–24.8 cmol_(+)_ kg^−1^), low available phosphorus (16.2–18.4 mg kg^−1^), medium exchangeable potassium (0.21–0.24 cmol_(+)_ kg^−1^), medium exchangeable calcium (3.52–3.71 cmol_(+)_ kg^−1^), low exchangeable magnesium (0.42–0.47 cmol_(+)_ kg^−1^), low available sulfur (5.47–5.81 mg kg^−1^), normal exchangeable aluminum content (0.21–0.28 cmol_(+)_ kg^−1^), high available copper (4.16–4.82 mg kg^−1^), high available zinc (3.51–3.97 mg kg^−1^), medium available manganese (5.68–8.92 mg kg^−1^), high available iron (141–162 mg kg^−1^), and medium boron available (0.29–0.38 mg kg^−1^).

A randomized complete block design with 4 blocks and 8 treatments per block was used, having a total of 32 experimental units per 40 m^2^. Treatments (T) consisted of 4 ages of cutting (30, 45, 60, and 90 days), with fertilization and without fertilization. In the fertilization treatments, 3400 kg fertilizer per hectare-year distributed according to the area and cutting day was applied, the day after each cut. Fertilizer nutrient concentration was 23.6% N, 12.5% P_2_O_5_, and 19.3% K_2_O. T1, T2, T3, and T4 were designated as the cuts after 30, 45, 60, and 90 days, respectively, with fertilization, and T5, T6, T7, and T8 were the cuts at 30, 45, 60, and 90 days, respectively, without fertilization. Moreover, in T1, T2, T3, and T4, 27.9, 41.9, 55.9 and 83.8 g m^2^/cut of fertilizer were applied, respectively.

Experimental data were taken after the second cut to have a stabilization period of the treatments and 4 experimental cuts were made for each treatment. The analyzed variables were biomass production, leaf-stem ratio, dry matter (DM), crude protein, neutral detergent fiber (NDF), acid detergent fiber (ADF), foliar content, and nutrient uptake of N, P, K, Ca, Mg, Na, Cu, Zn, Mn, Fe, B and nutrient recovery efficiency. Data were subjected to a variance analysis (ANOVA). When there were differences (P < 0.05), Duncan’s multi-range test was used^13^. In addition, a regression analysis was performed to determine the relationship between biomass production, leaf-stem ratio, DM, crude protein, NDF, ADF, with cutting days. Analyses were performed with SAS and MATLAB R2021a packages.

Samples for experimental analyses were taken from randomly determined areas per experimental unit per cut. For analysis of performance, 4 m^2^ were taken per experimental unit, each square meter was individually weighed, and the biomass production and leaf/stem ratio were determined. Approximately 4 kg were collected per experimental unit for bromatological analysis, nutrient concentration, and nutrient absorption. Once the sample was taken, it was weighed and dehydrated at 60 °C for subsequent analysis. Soil analyses were carried out in each experimental unit at the beginning of the experimental process. Bromatological and soil analyses were performed using the Weende and Van Soest methodology, and for N the Kjeldahl methodology was used. Moreover, K, Ca, Mg, Mn, Na, Fe, Zn, and Cu were analyzed using atomic absorption spectroscopy, P through UV–Vis spectroscopy (Bray II), B using UV–Vis spectroscopy (modified Olsen), and organic carbon by Walkey and Black methodology. The pH was studied by the potentiometric method, cation exchange capacity by sodium chloride (titration), and the interchangeable aluminum extracted with 1 N potassium chloride (titration). Nutrient recovery efficiency was calculated using the Eq. (1).

1 $$ {\text{Nutrient~recovery~efficiency}} = \frac{{N_{f}  - N_{w} }}{F} \times 100, $$

where N_f_ is the nutrient uptake in the treatment with fertilization, N_w_ is the nutrient uptake in the treatment without fertilization, F is the amount of nutrients added as fertilizer kg ha^–1^year^−1^.

## Results

**Figure 1:**
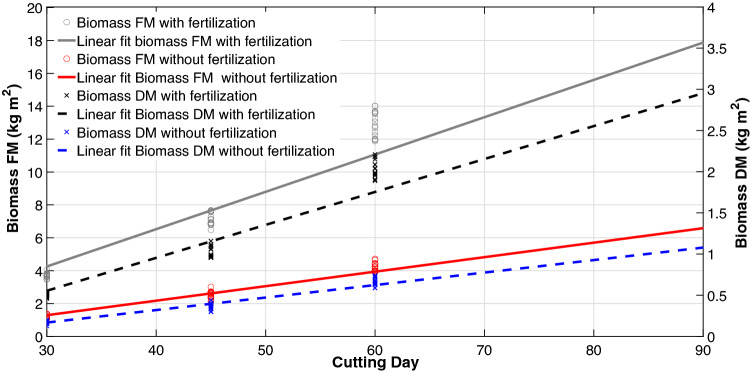
Linear relationship between biomass production of King grass on different cutting days and in systems with and without fertilization. Plots were created in MATLAB R2021a. *FM* fresh matter, *DM* dry matter. Linear regression equation (*C* cutting day): Biomass FM with fertilization = 0.2269 C − 2.5525, r^2^ = 0.9238, n = 63, P < 0.001. Biomass FM without fertilization = 0.0882 C − 1.352, r^2^ = 0.9784, n = 63, P < 0.001. Biomass DM with fertilization = 0.03998C − 0.6448, r^2^ = 0.9263, n = 63, P < 0.001. Biomass DM without fertilization = 0.01517 C − 0.2866, r^2^ = 0.9747, n = 63, P < 0.001.

The effect of fertilization and cutting days on biomass production are shown in Fig. 1. Biomass production increased linearly with fertilization and cutting days, the values ranging from 0.17 kg DM at 30 days to 1.07 kg DM at 90 days in treatments without fertilization and from 0.51 kg DM at 30 days to 2.84 kg DM at 90 days in treatments with fertilization.

**Figure 2:**
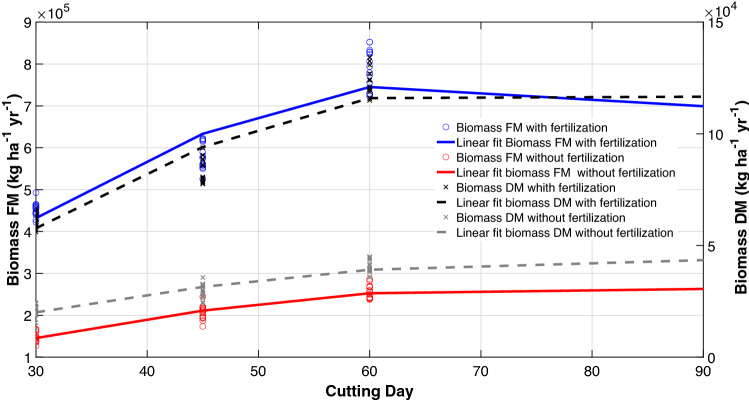
Linear relationship between biomass production of King grass on different cutting days and in systems with and without fertilization. Plots were created in MATLAB R2021a. *FM* fresh matter, *DM* dry matter. Lineal regression equations (*C* cutting day): Biomass FM with fertilization: − 199.4084 C^2^ + 28,382.8977C − 240,005.9343. r^2^ = 0.8113, n = 63, P < 0.001. Biomass FM without fertilization =  − 53.7566 C^2^ + 8406.3371 C − 58,284.7854. r^2^ = 0.9069, n = 63, P < 0.001. Biomass DM with fertilization =  − 32.0241 C^2^ + 4824.9404 C − 58,229.4563. r^2^ = 0.8290, n = 63, P < 0.001. Biomass DM without fertilization: =  − 8.2090 C^2^ + 1374.6121C − 13,776.6985. r^2^ = 0.8997, n = 63, P < 0.001.

Regarding biomass production per ha-year, there was a linear relationship with cutting days. In treatments with fertilization, King grass plants showed an accelerated development, mainly up to a cutting age of 45 days (T2), where the growth rate decreased until 60 days of cutting (T3), at which point biomass production was the highest (123,942 kg ha^−1^ year^−1^ DM) and has tended to stabilize (Fig. 2). Cuts at 30, 45 and 90 days showed productions of 61,679, 83,479 and 115,273 kg of DM, respectively.

In addition, it was determined that all fertilization treatments had higher values and were significantly different compared to unfertilized treatments (20,617, 29,936, 40,252, 43,262 kg DM for the 30 45 60 and 90 days, respectively), with increases up to sixfold in accumulated production. This indicated that King grass crops must be managed with fertilization systems.

### Bromatological composition of King grass

**Figure 3:**
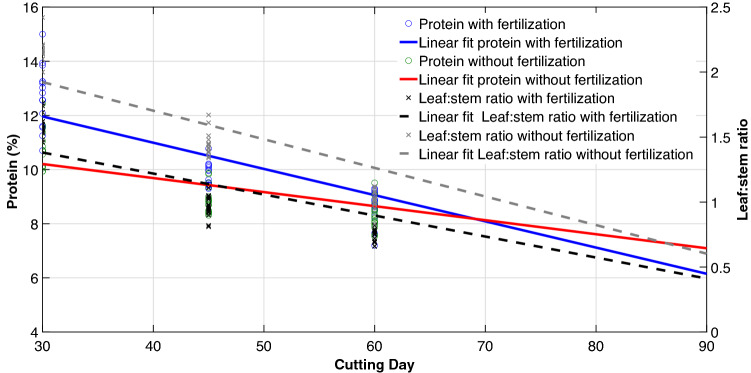
Linear relationship between protein and leaf: stem ratio of King grass on different cutting days and in systems with and without fertilization. Plots were created in MATLAB R2021a. Lineal regression equations (C = Cutting day): Protein with fertilization =  − 0.0968C + 14.8642. r^2^ = 0.8491, n = 63, P < 0.001. Protein without fertilization =  − 0.0519C + 11.7607. r^2^ = 0.7271, n = 63, P < 0.001. Leaf: stem ratio with fertilization =  − 0.0162C + 1.8657. r^2^ = 0.7986, n = 63, P < 0.001. Leaf: stem ratio without fertilization =  − 0.0220C + 2.5833. r^2^ = 0.8399, n = 63, P < 0.001.

Bromatological contents of King grass were statistically different (P < 0.01) in each analyzed variable. Crude protein showed a progressive linear reduction when cutting days increased (Fig. 3), being higher in T1 (12.80%). A greater reduction in protein production was observed when King Grass was fertilized, because of a less development in the crop, more tender and less developed plants and a greater leaf:stem ratio. The lowest value was found in T4 (6.53%), where higher plant development and lower leaf:stem ratio were found.

**Figure 4:**
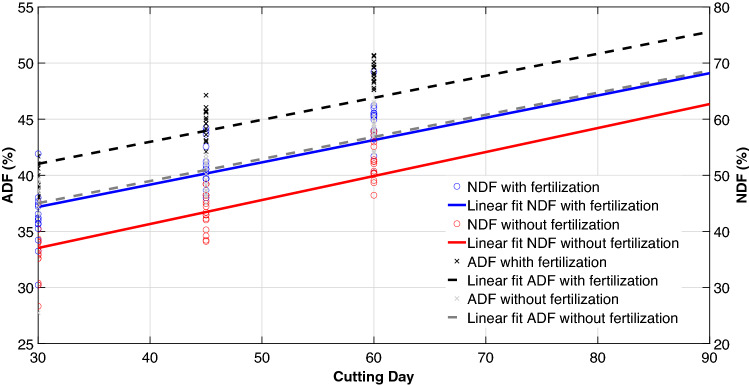
Linear relationship between neutral detergent fiber (NDF), acid detergent fiber (ADF) of King grass on different cutting days and in systems with and without fertilization. Plots were created in MATLAB R2021a. Lineal regression equations (*C* cutting day): Neutral detergent fiber with fertilization = 0.3922C + 40.2785. r^2^ = 0.8228, n = 63, P < 0.005. Neutral detergent fiber without fertilization = 0.3937C + 33.2255. r^2^ = 0.8383, n = 63, P < 0.005. Acid detergent fiber with fertilization = 0.1986C + 31.2225. r^2^ = 0.7877, n = 63, P < 0.001. Acid detergent fiber without fertilization = 0.2138C + 27.1088. r^2^ = 0.8436, n = 63, P < 0.001.

There was a linear increase in fiber with the age of cutting as a result of stem growth in treatments 2, 3, and 4; in treatments with fertilization, rapid plant development was observed (Fig. 4). The same trend was observed for treatments without fertilization but with lower values.

A reduction in the synthesis of protein compounds with age was related to the increase in structural carbohydrates demonstrated by negative correlations of 77% and 74% with NDF and ADF against protein contents. The leaf:stem ratio showed a 74% correlation with the protein. The results showed the significant effect of cutting age and fertilization on the concentration of nutrients in the plant and demonstrated the importance of establishing the best relationship between cuts and fertilization, based on the use for which the crop is established.

### Foliar content

Foliar contents showed statistical differences (P < 0.05) for all analyzed variables, except sodium (Na). Cutting age had a significant effect on mineral content; as the number of days increased, the plant´s mineral content was significantly reduced. Treatments with fertilization had a reduction in the concentration of minerals between 13.1 and 48.6% and treatments without fertilization between 9.7 and 31.3%. Elements incorporated into fertilization (N, P, K) had higher values on the same cutting days and showed increases of 17.9, 27.3, and 12.5% for N, P, and K respectively, demonstrating the effect of fertilization on the foliar contents of plants. Elements that were not incorporated into fertilization (Ca, Mg, Cu, Zn, Mn, Fe, and B) had higher values in non-fertilization treatments where the plant was less developed (Table 1).

**Table 1:** King grass foliar content based on cutting days and fertilization.

Treatment	N	P	K	Ca	Mg	Na	Cu	Zn	Mn	Fe	B
	%						mg kg^−1^				
T1	2.03^a^	0.28^a^	1.98^a^	0.41^bc^	0.23^ba^	0.086	18.28^b^	67.92^ba^	115.26^b^	99.25^bac^	138.22^a^
T2	1.56^c^	0.24^b^	1.91^a^	0.39^dc^	0.21^bac^	0.089	18.24^b^	62.98^bc^	109.25^b^	95.71^bc^	125.02^ba^
T3	1.39^d^	0.20^cd^	1.87^ba^	0.35^dc^	0.20^bc^	0.091	16.21^b^	55.96^dc^	105.88^b^	75.28^ed^	105.55^bc^
T4	1.04^f^	0.16^e^	1.72^dc^	0.30^d^	0.18^c^	0.085	15.56^b^	48.64^d^	78.69^c^	65.22^e^	90.85^c^
T5	1.72^b^	0.22^cb^	1.76^bc^	0.52^a^	0.24^a^	0.087	22.34^a^	74.24^a^	132.25^a^	113.57^a^	148.23^a^
T6	1.42^d^	0.22^cb^	1.71^dc^	0.50^ba^	0.22^ba^	0.0725	18.73^b^	70.32^ba^	114.33^b^	112.32^ba^	144.25^a^
T7	1.33^d^	0.19^cde^	1.68^dc^	0.45^bac^	0.22^ba^	0.081	16.94^b^	65.95^ba^	109.11^b^	100.21^bac^	122.52^ba^
T8	1.18^e^	0.17^de^	1.59^d^	0.44^bac^	0.20^bc^	0.083	16.01^b^	62.54^bc^	107.35^b^	88.46^dc^	124.03^ba^
CV	6.29	10.26	4.83	13.44	10.60	24.88	12.57	8.69	10.01	9.97	12.38

*CV* coefficient of variation.

Measures in columns followed by different letters are significantly different (P < 0.05), based on Duncan’s multi-range test.

### Nutrient uptake

Nutrient uptake per ha^−1^ year^−1^ showed statistical differences (P < 0.05) for all analyzed variables, with significant variations in fertilization and cutting days. The plant increased nutrient uptake up to 60 days in treatments with and without fertilization; in contrast, at 90 days, a decrease in nutrient uptake for all elements was observed and a reduction in nutrient production capacity was also observed. When fertilization was implemented, the grass showed a significant increase in its nutrient uptake capacity with absorption up to 3.4-fold higher. Although nutrient uptake in treatments without fertilization was lower, it remained high (534.6, 76.5, and 676.2 kg ha^−1^ year^−1^ for N, P, and K respectively) (Table 2), leading to continuous extraction of minerals from the soil and a loss of the soil’s ability to provide available and interchangeable nutrients.

**Table 2:** Nutrient uptake of King grass kg ha^−1^ year^−1^.

Treatment	N	P	K	Ca	Mg	Na	Cu	Zn	Mn	Fe	B
	kg ha^−1^ year^−1^										
T1	1253.3^b^	172.7^c^	1221.2^d^	252.9^d^	141.9^c^	53.0^c^	1.1^c^	4.2^c^	7.1^c^	6.1^c^	8.5^b^
T2	1305.6^b^	200.4^b^	1594.5^c^	325.6^c^	175.3^cb^	74.3^b^	1.5^b^	5.3^b^	9.1^b^	8.0^ba^	10.4^b^
T3	1720.3^a^	247.9^a^	2317.7^a^	433.8^a^	247.9^a^	112.8^a^	2.0^a^	6.9^a^	13.1^a^	9.3^a^	13.1^a^
T4	1203.5^b^	184.4^cb^	1982.7^b^	354.8^b^	207.5^b^	98.0^a^	1.8^ba^	5.6^b^	9.1^b^	7.5^b^	10.5^b^
T5	355.4^d^	45.4^e^	362.9^f^	107.2^g^	49.5^e^	17.9^d^	0.5^d^	1.5^e^	2.7^e^	2.3^e^	3.1^d^
T6	426.3^dc^	65.9^ed^	511.9^fe^	149.7^fg^	65.9^ed^	21.7^d^	0.6^d^	2.1^ed^	3.4^ed^	3.4^ed^	4.3^dc^
T7	534.6^c^	76.5^d^	676.2^e^	181.1^fe^	88.6^d^	32.6^d^	0.7^d^	2.7^d^	4.4^ed^	4.0^d^	4.9^dc^
T8	512.2^c^	73.6^d^	687.9^e^	190.3^e^	86.5^d^	35.9^dc^	0.7^d^	2.7^d^	4.6^d^	3.8^d^	5.4^c^
CV	9.71	12.04	10.90	20.15	17.0	26.00	15.79	11.25	15.30	15.12	16.11

*CV* coefficient of variation.

Measures in columns followed by different letters are significantly different (P < 0.05), based on Duncan's multi-range test.

### Nutrient recovery efficiency

**Figure 5:**
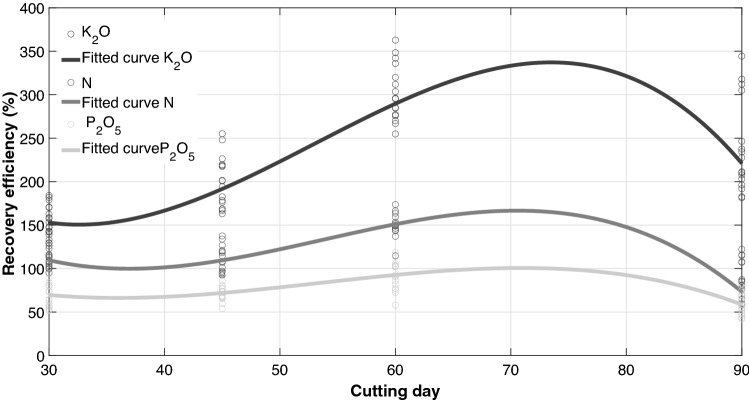
Cubic relationship between nutrient recovery efficiency and cutting day for (a) N (r^2^ = 0.903, n = 42, P < 0.001), (b) P_2_O_5_ (r^2^ = 0.6763, n = 43, P < 0.001), (c) K_2_O (r^2^ = 0.7803, n = 46, P < 0.001). Plots were created in MATLAB R2021a.

Nutrient recovery efficiency showed a progressive increase with the cutting days until treatment 3 where the highest efficiency was observed, followed by a decrease (Fig. 5). Efficiency ranged from 86.15% to 147.78% for N, from 59.79% to 92.42% for P_2_O_5_ and from 157.59% to 301.36% for K_2_O. This indicates that, under the same conditions of this study, cuts should be made between 60 and 70 days. A surprising plant response to fertilization and an interesting plant-soil-fertilizer interaction were also observed, which led to an incredible ability to take advantage of the nutrients in fertilizers and a significant increase in the absorption of nutrients from the soil. It is possible that this behavior is caused by a greater root development and a higher rate of soil nutrients mineralization, as a result of an increasing in microbial activity stimulated by root exudated and nutrient inputs in fertilization, taking into account that we worked with a soil rich in organic matter.

## Discussion

Martínez and González evaluated six varieties of *Pennisetum purpureum*, applied 250 kg of urea per ha^−1^ year^−1^, finding biomass productions between 14.1 and 38.8 t of DM ha^−1^ year^14^. Luna in a study about King grass production in different cutting ages (45, 60, 75, and 90 days), reported biomass productions of 5.88, 8.08, 10.68, and 13.42 ton of DM cut ha^−1^, respectively^3^. Uvidia^15^ obtained the same trend with cuts at 30, 45, 60, 75, and 90, reporting equally ascending values between 5 and 19 t ha^−1^ year^−1^. Data similar to those were found in non-fertilization treatments in this study, but significantly higher in treatments with fertilization. The high biomass production in the systems with fertilization gives King grass an important potential in the development of animal feeding systems, production of biofuels, composts and substrate for biodigestion, among others.

Luna^3^, in a study about King grass production with different cutting ages reported a leaf:stem ratio of 0.93, 0.85, 0.76, and 0.71 at 45, 60, 75 and 90 cutting days, respectively. Although they observed smaller variation than the ratio found in this study, the same trend was observed. Several authors concluded that this behavior was due to the maturity of the plant and its productive characteristics^16,17^.

Luna also reported crude protein contents between 7.93 and 9.41, where the lowest value was obtained for a higher cutting age^3^. These values were similar to those found in this study, indicating that nutritional quality in King grass is reduced with cutting age, while structural carbohydrates are significantly increased, which has been related to the increase in cellulose and hemicellulose production. Uvidia found the same trend in the relationship between protein and fiber content^15^.

Martínez and González, in a study of *Pennisetum purpureum* with different cutting days, found a protein production per year between 3 and 3.7 t without statistical differences^14^. In this work, the production of protein per hectare year ranged between 2.22 and 10.75 t ha^−1^ year^−1^ and the higher value was found in T3 where cuts were made at 60 days with fertilization. Martínez and González also found Ca concentrations between 0.21 and 0.59% and P concentrations between 0.24 and 0.46%, values similar to those found in this study for the various treatments^14^.

Asmare^12^ determined the effect of cutting age on mineral content in *Pennisetum pedicellatum* and found a reduction in mineral content, however, only Mg and Ca were significant (*P* < 0.05) with contents between 0.44 and 0.31% for Ca, similar to those found in this study, and contents between 398 and 278 mg kg^−1^ for Fe, which were higher than those found in this study. Zn contents ranged between 19.3 and 17.2 mg kg^−1^, which were lower than those found in this study. Similarly, Mn was between 54.0 and 80.2 mg kg^−1^, lower than those found here, except in treatment with fertilization and cutting at 90 days. Mg was between 0.52 and 0.36% higher than those found here, demonstrating that there is an important effect of cutting age and soils on nutrient concentration in plants.

King grass has low nutritional contents (low protein and high fiber contents) compared to high nutritional quality grasses such as *Pennisetum clandestinum* (21.9%, 62.2% and 27.4% of crude protein, NDF and ADF respectively^18^), and *Lolium perenne* (17.6%, 36 and 22 of crude protein, NDF and ADF respectively^19^). However, its high biomass production and carrying capacity (25.5 livestock units per hectare, at T6) makes it a plant with a great potential to increase productivity and land use. The use of King grass in systems combined with forage plants of high nutritional quality (*Tithonia diversifolia*, *Morus alba*, *Moringa oleifera*, *Alocasia macrorrhiza*, *Sambucus nigra*, *Boehmeria nivea*, among others) or its establishment in farms of small- and medium-sized beef cattle producers would allow achieving production models with high carrying capacity and adequate nutritional intake.

Permanent cutting systems without fertilization lead to continuous nutrient uptake, resulting in a progressive loss in the soil's ability to provide nutrients to the plant and, consequently, a progressive reduction in biomass and nutrient production. Additionally, the application of fertilizers (NPK) stimulates the absorption of other nutrients, achieving a more than twofold increase in absorption after 60 days compared to treatments without fertilization. This guarantees high yields, but could also lead to soil fertility losses. For this reason, fertilizers should be formulated based on soil analysis and plant nutritional requirements (Table 2) to promote higher productivity and soil sustainability.

Knowledge of plant nutritional requirements and development of fertilization systems based on these requirements ensures maximum crop productivity and sustainability over time^20–22^. Singh, in an experiment about the effect of nutrient omissions in wheat, found that maximum productivity and nutritional quality was achieved when a balanced mix of fertilizers (N, P, K, and S) was applied, the omission of a nutrient had a direct effect by reducing concentrations and absorption of these elements in the crop^23^.

Adeniyan studied the effect of the combination of N, P, and K on nutrient absorption and corn yield using a granular and foliar fertilizer in doses of 100, 80, 60, and 40 kg ha^−1^; the highest values of N, P, and K uptake were 144.2, 53.5, and 142.6 kg ha^−1^, respectively, and the lowest values were 60.5, 14.4, and 63.4 kg ha^−1^, respectively^24^. Rosado, in a study on nutrient absorption, evaluated the effect of the application of three nitrogen fertilizers and six nitrogen doses (0, 120, 240, 360, 480, and 600 kg ha) on *Panicum maximum* cv. ‘Mombasa’ macronutrient uptake and found that nutrient absorption had a significant increase with fertilization; N absorption increased 320 kg ha^−1^ year^−1^, and the same trend was found for P and K^25^. Those results were similar to those found in this study with an effect of fertilization on the absorption capacity and nutrient production of plants.

Studies carried out in different crops (broccoli, potato, lettuce, corn, wheat, rice) in which nutrient recovery efficiency was determined, the highest efficiency reported for nitrogen, phosphorus and potassium was 91%, 38% and 88% respectively^26–32^; these values were lower than those found in this study, demonstrating again the very high capacity of the plant to take advantage of nutrients from fertilizers and soil and the importance of studying and understanding the mechanisms used by the plant to achieve these efficiencies.
